# Long-term effectiveness of levodopa-carbidopa intestinal gel on motor and non-motor symptoms in advanced Parkinson’s disease: results of the Italian GLORIA patient population

**DOI:** 10.1007/s10072-020-04401-w

**Published:** 2020-04-28

**Authors:** Angelo Antonini, Pietro Marano, Graziano Gusmaroli, Nicola Modugno, Claudio Pacchetti, Mariachiara Sensi, Gabriella Melzi, Lars Bergmann, Maurizio Zibetti, Leonardo Lopiano

**Affiliations:** 1 Parkinson and Movement Disorders Unit, Department of Neuroscience, University of Padua, Padua, Italy; 2 Madonna del Rosario Clinic, Catania, Italy; 3 Neurology Unit, Ospedale degli Infermi, Biella, Italy; 4 IRCCS Neuromed, Pozzilli, Italy; 5 Parkinson’s Disease and Movement Disorders Unit, IRCCS C. Mondino Foundation, Pavia, Italy; 6 Neurology Unit, Sant’Anna University Hospital, Ferrara, Italy; 7 Medical Department, AbbVie s.r.l., Campoverde di Aprilia, Italy; 8 AbbVie Inc., North Chicago, IL USA; 9 Department of Neuroscience, University of Torino, AOU Città della Salute e della Scienza, Torino, Italy

**Keywords:** Parkinson’s disease, Levodopa-carbidopa intestinal gel, Motor symptoms, Non-motor symptoms, Quality of life, Routine patient care

## Abstract

**Introduction:**

The GLORIA registry included 375 advanced Parkinson’s disease (PD) patients and evaluated the efficacy and safety of a 24-month levodopa-carbidopa intestinal gel (LCIG) treatment in routine medical care. This analysis focuses on the Italian population, 60 patients treated with LCIG in 7 specialised PD care centres.

**Methods:**

Hours of “Off” and “On” time were assessed with a modified version of the Unified Parkinson’s Disease Rating Scale (UPDRS) part IV items 39 and 32. Motor fluctuations, dyskinesia, non-motor symptoms, quality of life and safety were evaluated.

**Results:**

Overall, 42 (70%) out of 60 patients completed the registry. LCIG treatment reduced “Off” time (− 3.3 ± 2.7 h at month 24 (M24), *P* < 0.0001), increased “On” time with dyskinesia (− 2.6 ± 5.2 h at M12, *P* = 0.0160), and improved UPDRS II and UPDRS III total scores at M24 (− 4.5 ± 10.6, *P* = 0.0333 and − 4.9 ± 11.7, *P* = 0.0229, respectively), Non-Motor Symptom Scale (NMSS) total score (− 21.8 ± 28.5, *P* < 0.0001) and Parkinson’s Disease Questionnaire-8 item (PDQ-8) total score (− 12.5 ± 23.9, *P* = 0.0173) versus previous oral therapy. Adverse drug reactions (ADR) possibly or probably related to treatment were reported in 16 (28.6%) patients. Decreased weight (7.1%), polyneuropathy (7.1%) and abdominal pain (5.4%) were the most frequent ADRs while device malfunction (5.4%) and medical device change (5.4%) were the most reported device complaints.

**Conclusions:**

LCIG improved motor fluctuations, non-motor symptoms and quality of life over 24 months while tolerability was consistent with the established safety profile.

## Introduction

Parkinson’s disease (PD) is a progressive neurodegenerative disorder, and levodopa is affirmed as the most effective drug for treatment [1]. PD in advanced stage and long-term oral levodopa administration may account for disabling motor complications [2, 3]. Although primarily considered as a movement disorder, PD patients present a broad range of non-motor symptoms (NMS) [4, 5], significantly contributing to co-morbidities and loss of autonomy, leading to a decline of quality of life (QoL) [6] and increase in PD-related health care costs [7, 8].

As PD progresses, the duration of the levodopa response shortens and the therapeutic window narrows. Fluctuating peripheral levodopa plasma levels, caused, e.g. by erratic gastric emptying, increasingly provoke motor fluctuations [9–11] and severely interfere with daily activities, social interactions and patient’s quality of life (QoL) [12, 13].

Levodopa-carbidopa intestinal gel (LCIG) is continuously released to the upper intestine and helps to achieve more stable levodopa plasma levels compared to oral levodopa administration [14, 15]. As a consequence, motor fluctuations decrease and non-motor symptoms [16–20] and QoL improve [12, 13].

To date, only a few studies evaluated the long-term efficacy and safety of LCIG treatment in routine clinical care in a large cohort of advanced PD patients [8, 18, 21–24]. The GLORIA registry systematically assessed the long-term effectiveness of LCIG on motor symptoms as well as on non-motor symptoms (NMS) and QoL; in addition, safety of LCIG was evaluated.

Due to the observational nature of the GLORIA registry reflecting the routine clinical care for LCIG management, the aim of this paper was to assess the outcomes of the Italian patient cohort treated in 7 specialised PD care centres and to detect differences with the results of the overall PD population included in GLORIA study across 18 countries [25, 26].

## Methods

### Study design

Seventy-five specialised PD care unit centres across 18 countries included 375 patients with advanced PD experiencing motor complications in this 24-month multi-national, non-interventional, observational registry. According to the standard medicinal product characteristics, the PD patient and the treating neurologist should jointly decide to switch to LCIG treatment when oral and transdermal treatments cannot be further optimised. Clinical outcomes were recorded every 6 months for the initial 24-month LCIG treatment. The study protocol was approved by health authorities and national and/or local independent ethics committees in each country and at each participating centre.

The outcomes of the 12-month interim and the final 24-month analysis of the GLORIA registry were published [25, 26]. This paper presents the results of the Italian study population, 60 patients treated with LCIG at the 7 Italian PD care unit centres.

For statistical analyses, ANOVA over time and paired *t* tests were performed for the comparison of all efficacy outcomes to baseline (BL).

### Patients

All patients provided written informed consent prior to inclusion. In LCIG-naïve patients (63%), all observations were recorded prospectively. Some of the patients received LCIG for ≤ 12 months before inclusion, and clinical data were collected retrospectively up to enrolment and were recorded thereafter prospectively as of the M12 to the M24 follow-up. LCIG treatment was initiated in the majority of patients by using a temporary nasojejunal (NJ) tube to titrate and optimise the dose before being administered through percutaneous endoscopic gastrostomy with  jejunal extension (PEG-J) (according to local label and reimbursement criteria).

### Efficacy

The actual hours of “Off” time and “On” time with dyskinesias were assessed using the Unified Parkinson’s Disease Rating Scale IV (UPDRS) Items 39 and 32 modified according to the Movement Disorder Society (MDS)-UPDRS (corresponding parts 4.3 and 4.1). The UPDRS parts II, III, and IV were assessed in the “On” state. Non-motor symptoms were evaluated using the NMSS and patient-reported quality of life (QoL) measures included the disease-specific PDQ-8. Efficacy assessments were collected at baseline (BL) before LCIG treatment initiation, at discharge from hospital following PEG-J placement (day 1-D1), at month 6 (M6), M12, M18, and M24. All outcomes at follow-up visits were analysed as mean change from baseline.

### Safety

Adverse drug reactions (ADRs), considered by the investigator as adverse events with a reasonable possible causal relationship to the treatment drug or the device, were recorded and coded according to the Medical Dictionary for Regulatory Activities (MedDRA) [27] and categorised by the study investigator as mild, moderate, or severe, with unlikely, possible of probable relationship to the drug/device system. Serious ADRs and product complaints were monitored and recorded.

## Results

### Patient population, demographics, and disease characteristics at baseline

Out of the 60 patients enrolled, 42 (70%) completed the registry. Patient demographics, PD characteristics, and baseline assessments of motor symptoms, NMSS, and QoL are summarised in Table 1. Reasons for premature discontinuation (*N* = 15, 25%) were withdrawal of consent (*N* = 7, 4 withdrawals between BL and D1 visit), lack of efficacy (*N* = 3), adverse events (ADRs, concomitant diseases, or death; *N* = 2), protocol violations (*N* = 2), and administrative reasons (*N* = 1). No return to follow-up visits was reported for 3 (5%) patients.

**Table 1:** Demographics, medical history, disease characteristics and previous PD treatments recorded at baseline (BL) presented in mean ± SD or number (%)

Gender^a^	Male	37 (61.7)
	Female	23 (38.3)
Age (years)^a^		68.3 ± 8.1
Time since PD diagnosis (years)^a^		11.7 ± 5.8
Hoehn and Yahr^b^		
During “On”		2.7 ± 0.6
During “OFF”		3.6 ± 0.8
UPDRS Part IV^b^		
Modified item 39: “Off” phase (h/day)		4.3 ± 2.7
Modified item 32: dyskinesia (h/day)		4.7 ± 4.1
UPDRS Part II (activities of daily living)^b^ “On”		18.9 ± 10.4
UPDRS Part III (motor examination)^b^ “On”		29.4 ± 10.8
NMSS total score^b^		66.0 ± 45.1
PDQ-8 total score^b^		52.6 ± 21.7
EQ-5D score^b^		0.33 ± 0.35
EQ-VAS score^b^		45.8 ± 23.4
PD medications reported at BL		
Levodopa		57 (95.0)
Total daily oral dose (mg)		846 ± 361
Dopamine agonist		44 (73.3)
COMT inhibitors		31 (51.7)
MAO-B inhibitors		12 (20.0)
Amantadine		11 (18.3)
Other oral		9 (15.0)

*PD* Parkinson’s disease, *UPDRS* Unified Parkinson’s Disease Rating Scale, *NMSS* non-motor symptom scale, *PDQ-8* Parkinson’s Disease Questionnaire 8-item, *EQ-5D* Euro Quality of Life 5 Dimensions, *EQ-VAS* Euro Quality of Life Visual Analog Scale, *COMT* catechol-O-methyltransferase, *MAO-B* monoamine oxidase-B

^a^All subjects consented population (*N* = 60)

^b^Full analysis set population (*N* = 52)

### Treatments

The most common concomitant medications across all study visits were oral levodopa and dopamine agonists and catechol-O-methyltransferase (COMT) inhibitors (Table 1). Among patients who received LCIG as a monotherapy, the mean daily levodopa equivalent dose (LED) [28] ranged from 1278 ± 675 mg at D1 (*n* = 19) to a maximum of 1456 ± 529 mg at M12 (*n* = 15) and to 1396 ± 585 mg at M24. The mean daily LED for patients with LCIG combination therapy ranged from 1814 ± 744 mg at D1 (*n* = 36) to a maximum of 1847 ± 744 mg at M6 (*n* = 30) and to 1845 ± 713 mg at M24.

### Efficacy

**Fig. 1:**
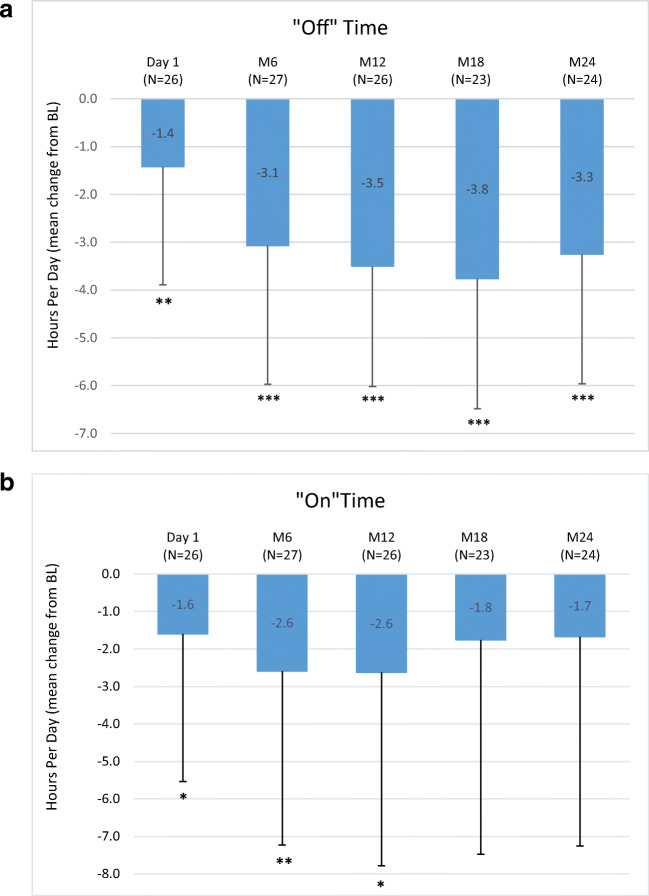
Motor symptom efficacy: mean change from BL of daily hours of **a** “Off” time (modified UPDRS Part IV Items 39) and **b** “On” time with dyskinesia (modified UPDRS Part IV Item 32) at start of LCIG treatment with permanent tube (D1), at M6, M12, M18 and M24 compared to BL in a paired *t* test at the *P* < 0.05 (*), *P* < 0.01 (**) and *P* < 0.001 (***). Numbers indicated in brackets represent the numbers of matched pairs. Error bars indicate SD. UPDRS = Unified Parkinson’s Disease Rating Scale; BL = baseline; D1 = discharge from hospital post-PEG-J placement; M = month

**Fig. 2:**
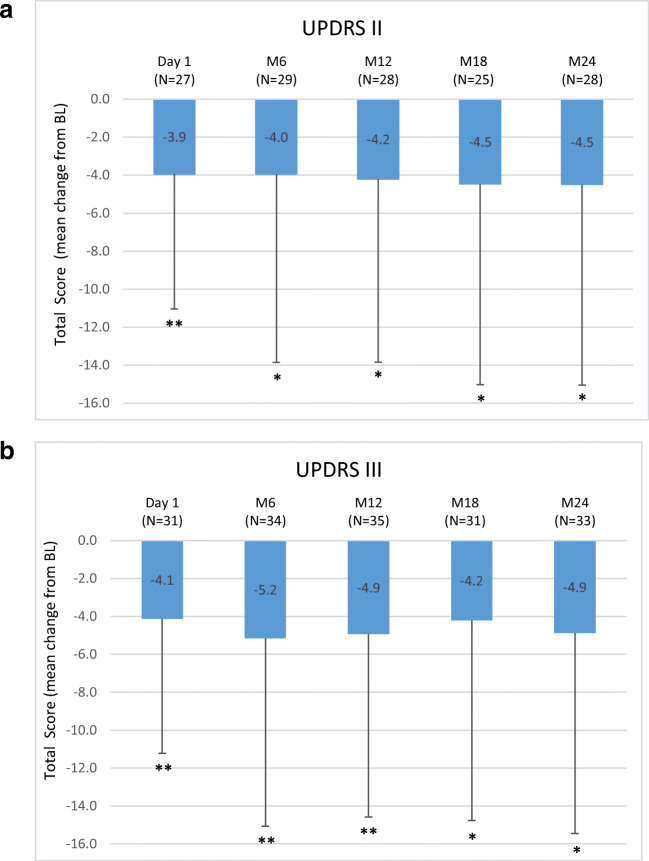
Motor symptoms **a** of UPDRS II and **b** UPDRS III scores at start of LCIG treatment with permanent tube (D1), at M6, M12, M18 and M24 compared to BL in a paired *t* test at the *P* < 0.05 (*), *P* < 0.01 (**) and *P* < 0.001 (***). Numbers indicated in brackets represent the numbers of matched pairs. Error bars indicate SD. UPDRS = Unified Parkinson’s Disease Rating Scale; Part II (activities of daily living) and Part III (motor examination); BL = baseline; D1 = discharge from hospital post-PEG-J placement; M = month

**Fig. 3:**
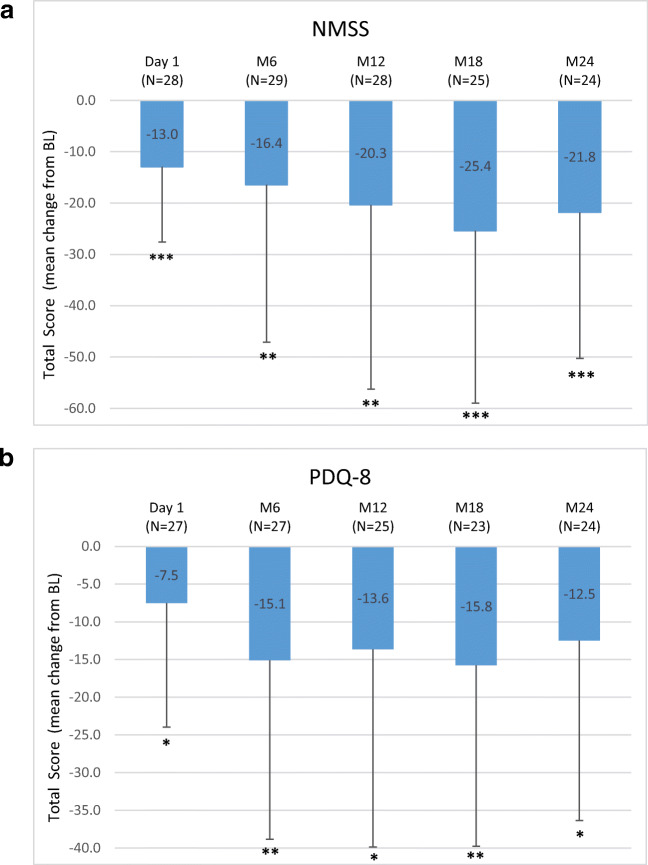
**a** Non-motor symptom improvements (NMSS total score reduction) and **b** quality of life improvement (PDQ-8 score reduction) at start of LCIG treatment with permanent tube (D1), at M6, M12, M18 and M24 compared to BL in a paired *t* test at the *P* < 0.05 (*), *P* < 0.01 (**) and *P* < 0.001 (***). Numbers indicated in brackets represent the numbers of matched pairs. Error bars indicate SD. NMSS = non-motor symptom scale, PDQ-8 = Parkinson’s Disease Questionnaire 8-item; BL = baseline; D1 = discharge from hospital post-PEG-J placement; M = month

A significant reduction from BL for the mean (±SD) number of “Off” hours was observed through the entire study period. Results at D1 (− 1.4 ± 2.5 h; *P* = 0.0070) and M24 (− 3.3 ± 2.7 h; *P* < .0001) versus BL are reported. Moreover, compared to BL, the mean “On” time with dyskinesia showed a significant improvement at D1 (− 1.6 ± 3.9 h; *P* = 0.0491), M6 and M12 (− 2.6 ± 5.1 h; *P* = 0.0160) (Fig. 1a and b). UPDRS II and III scores (assessed when “On”) showed significant reductions compared to BL through M24 (− 4.5 ± 10.6; *P* = 0.0333 and − 4.9 ± 11.7; *P* = 0.0229), respectively (Fig. 2a and b). The NMSS total score was significantly reduced compared to BL at all study visits with a maximum mean change of − 25.4 ± 33.6 at M18 (*P* = 0.0009) (Fig. 3a). The PDQ-8 total score was significantly reduced at every study visit (− 12.5 ± 23.9 at M24; *P* = 0.0173) (Fig. 3b).

### Safety

Overall, 16 (28.6%) patients experienced one or more ADRs (Table 2). The most frequently reported ADRs were decreased weight (7.1%), polyneuropathy (7.1%) and abdominal pain (5.4%), while device malfunction (5.4%) and medical device change (5.4%) were the most frequent device complaints. These ADRs were generally transient. Serious and severe ADRs were reported in 17.9% and 10.7% patients, respectively, and one patient (1.8%) with an ADR leading to LCIG discontinuation was reported. Two deaths occurred during the registry period, one for myocardial infarction and one for pulmonary oedema, both deemed as unlikely related by the investigator’s judgement.

**Table 2:** Tolerability of LCIG infusion: overall summary of ADRs/product complaints reported during LCIG infusion with temporary NJ tube and permanent PEG/J, ADRs reported with an incidence of ≥ 3% and all serious ADRs (% of *N* = 56)

Patients with at least one ADR*	16 (28.6)
Patients with at least one possibly or probably related*	13 (23.2)
Patients with at least one serious*	10 (17.9)
Patients with at least one severe*	6 (10.7)
Patients with at least one leading to LCIG discontinuation*	1 (1.8)
ADRs or product complaints occurring in ≥ 3% of patients^a^	
*Weight decreased*	*4 (7.1)*
Polyneuropathy	4 (7.1)
*Abdominal pain*	*3 (5.4)*
*Device malfunction*	*3 (5.4)*
*Medical device change*	*3 (5.4)*
*Gastrointestinal stoma complication*	*2 (3.6)*
Delirium	2 (3.6)
Serious ADRs or product complaints occurring in ≥ 1% of patients^b^	
*Pneumoperitoneum*	2 (3.6)
*Delirium*	*2 (3.6)*
*Abdominal pain*	*1 (1.8)*
*Ileus*	1 (1.8)
Dyskinesia	1 (1.8)
Polyneuropathy	1 (1.8)
Somnolence	1 (1.8)
Anaemia	1 (1.8)
Myocardial infarction	1 (1.8)
*Device malfunction*	*1 (1.8)*
Pneumonia	1 (1.8)
*Gastrointestinal stoma complication*	1 (1.8)

Note: “*” Denominator is count of patients in the regarding population

Gastrointestinal and gastrointestinal procedure-related ADRs are italicised

^a^Data indicates incidence of ADRs

^b^During 24 months of LCIG infusion via PEG-J

*ADR* adverse drug reaction (adverse events with a possible/probable relationship to the treatment drug or device), *GI* gastrointestinal, *LCIG* levodopa-carbidopa intestinal gel, *PEG-J* percutaneous endoscopic gastrostomy with jejunal extension

## Discussion

The GLORIA registry is one of the largest cohorts of advanced PD patients treated with LCIG and provided real-life evidence for the long-term effectiveness in reducing motor fluctuations and dyskinesia during routine clinical care. In addition, marked improvements in non-motor symptoms and QoL were demonstrated over the 24-month treatment period.

The analysis of the Italian sub-population consisting of 60 patients included by 7 specialised PD care centres in Italy revealed a significant reduction of the time patients spent in the “Off” condition. Patients treated with LCIG experienced a 57%, 87**%** and 75% reduction from BL in “Off” time at day 1, M18 and M24, respectively. This reduction in “Off” time was well above what is deemed clinically relevant [29] and was consistent with or even better than published open-label studies and randomised controlled trials on LCIG [8, 16–26] and higher compared to a 65% reduction in “Off” time at M24 demonstrated in the GLORIA registry [26]. In fact, in this sub-population analysis, we found a 77% reduction of the mean daily “Off” time compared to baseline.

The **“**On**”** time with dyskinesia was significantly reduced from BL in patients treated with LCIG by 34% and 56**%** at D1 and M12, respectively. The magnitude of reduction was similar at M18 and M24, however not significant, most likely due to the small number of observations. These outcomes reflect the current opinion that switching to continuous levodopa delivery not only improves motor fluctuations, but also reduces pre-existing dyskinesia. In the GLORIA population, the average “On” time with dyskinesia was reduced by 25**%** at all visits [26]. The marked reduction in “OFF” time and parallel substantial decrease in “On” time with dyskinesia at day 1 were achieved by switching from oral PD medications to LCIG. Importantly, these improvements in “OFF” time and “On” time with dyskinesia were obtained maintaining a stable LED throughout the 24-month LCIG treatment, reflecting experience in using LCIG and good treatment follow-up of patients. In contrast, the LED increased slightly in the total GLORIA population [26]. In this registry, no specific recommendations were provided for optimisation of oral doses prior to switching to LCIG and titration of LCIG during the initiation of infusion. The applied individual procedures reflected the ‘real-world’ routine clinical care. Treatment with LCIG led to a significant 20% to 38% reduction in NMSS score throughout the study which together with motor improvement resulted in a significant 14% to 30% reduction of the PDQ-8 score throughout the 24-month treatment [25, 26]. Also, the percentage reductions of NMS and PDQ-8 vs BL were greater in the Italian sub-group analysis compared to the total GLORIA population even if the Italian group baseline “Off” time was lower (4.3 ± 2.7 vs 6.0 ± 3.2 h). This observation seems of interest considering that in another GLORIA sub-analysis, it was recently reported that the magnitude of QoL improvement was greater in patients with more “Off” time and larger LED dose at baseline [30]. LCIG led to significant improvements in UPDRS II and III scores, 24% and 17%, respectively, at M24. These data are similar to those reported in previous studies examining LCIG treatment [2, 6, 8, 9, 23] and better to the one reported in the GLORIA population [26].

Since only ADRs, considered by the investigator as adverse AEs with a reasonable causal relationship to the treatment (drug/device), were recorded in GLORIA with less intense monitoring compared to clinical studies, the safety analyses of this registry cannot be directly compared with the results of clinical studies. Furthermore, the safety outcomes of the Italian cohort compared to the GLORIA population were different, most likely due to local reporting procedure and other factors. In the Italian cohort, only 29% of the patients reported ADRs, while in the GLORIA population, 55% of the patients reported ADRs. Device and procedure-related events were the most frequently reported ADRs in the GLORIA population, yet were less frequently reported in the Italian cohort. These ADRs were generally transient, occurring mainly within the first month post-PEG-J placement. This outcome emphasises the importance for a close collaboration between the neurologist and gastroenterologist during the PEG-J placement and the need for an intense monitoring in the immediate post-PEG-J placement period. Also, this may reflect the long-standing experience of the seven specialised Italian PD care centres. Overall, the most frequently reported ADRs and the GI-related ADRs were consistent with the known long-term complications of the PEG-J procedure [31] and the established safety profile of LCIG [16–18, 32].

Considering the mean age (68 years) and disease characteristics of the Italian patient population, LCIG procedures and treatment were generally well tolerated, with a lower rate of discontinuation due to ADR (1.8%) compared to a reported rate for the GLORIA population (7%) [26]. Out of the two deaths occurring during the registry, one was not related to treatment (myocardial infarction) and one unlikely related (pulmonary oedema).

This analysis provides an important “real life” insight in the LCIG treatment of advanced PD patients and revealed some specific facets on LCIG experience and treatment modalities in Italy. However, the design of this registry includes some limitations, such as the registry’s open-label design, the lack of a control group, not allowing to compare efficacy and safety assessments, and a partially retrospectively collection of data in some of the patients up to M12, while in all naïve LCIG patients (63%), all data were collected prospectively. However, according to the pre-defined sub analysis of retro- versus prospective data collection, there were no differences in the total population. In addition, a potential variability of data could be due to the broad geographical distribution of the participating sites in 18 countries, with the potential for different treatment approaches [25, 26]. The latter was addressed with this analysis of the Italian population comparing the results with the overall GLORIA outcomes.

In conclusion, LCIG resulted in sustained reductions over the 24-month treatment period in motor fluctuations and NMS burden and improvement of QoL, in advanced PD patients in routine care in Italian PD care centres. The tolerability of LCIG was consistent with the previously established safety profile.
